# Multiscale Computer Modeling of Spreading Depolarization in Brain Slices

**DOI:** 10.1523/ENEURO.0082-22.2022

**Published:** 2022-08-18

**Authors:** Craig Kelley, Adam J. H. Newton, Sabina Hrabetova, Robert A. McDougal, William W Lytton

**Affiliations:** 1 Program in Biomedical Engineering, SUNY Downstate Health Sciences University & NYU Tandon School of Engineering, Brooklyn, NY, 11203; 2 Department of Physiology and Pharmacology, SUNY Downstate Health Sciences University, Brooklyn, New York 11203; 3 Department of Cell Biology, SUNY Downstate Health Sciences University, Brooklyn, New York 11203; 4 Robert F. Furchgott Center for Neural and Behavioral Science, SUNY Downstate Health Sciences University, Brooklyn, New York 11203; 5 Department of Biostatistics, Yale University, New Haven, Connecticut 06513; 6 Yale Center for Medical Informatics, Yale University, New Haven, Connecticut 06513; 7 Program in Computational Biology and Bioinformatics, Yale University, New Haven, Connecticut 06513; 8 Department of Neurology, SUNY Downstate Health Sciences University, Brooklyn, New York 11203; 9 Department of Neurology, Kings County Hospital Center, Brooklyn, New York 11203

**Keywords:** extracellular space, hypoxia, NEURON, reaction-diffusion, spreading depolarization, spreading depression

## Abstract

Spreading depolarization (SD) is a slow-moving wave of neuronal depolarization accompanied by a breakdown of ion concentration homeostasis, followed by long periods of neuronal silence (spreading depression), and is associated with several neurologic conditions. We developed multiscale (ions to tissue slice) computer models of SD in brain slices using the NEURON simulator: 36,000 neurons (two voltage-gated ion channels; three leak channels; three ion exchangers/pumps) in the extracellular space (ECS) of a slice (1 mm sides, varying thicknesses) with ion (K^+^, Cl^–^, Na^+^) and O_2_ diffusion and equilibration with a surrounding bath. Glia and neurons cleared K^+^ from the ECS via Na^+^/K^+^ pumps. SD propagated through the slices at realistic speeds of 2–4 mm/min, which increased by as much as 50% in models incorporating the effects of hypoxia or propionate. In both cases, the speedup was mediated principally by ECS shrinkage. Our model allows us to make testable predictions, including the following: (1) SD can be inhibited by enlarging ECS volume; (2) SD velocity will be greater in areas with greater neuronal density, total neuronal volume, or larger/more dendrites; (3) SD is all-or-none: initiating K^+^ bolus properties have little impact on SD speed; (4) Slice thickness influences SD because of relative hypoxia in the slice core, exacerbated by SD in a pathologic cycle; and (5) SD and high neuronal spike rates will be observed in the core of the slice. Cells in the periphery of the slice near an oxygenated bath will resist SD.

## Significance Statement

Spreading depolarization (SD) is a slow-moving wave of electrical and ionic imbalances in brain tissue and is a hallmark of several neurologic disorders. We developed a multiscale computer model of brain slices with realistic neuronal densities, ions, and oxygenation. Our model shows that SD is exacerbated by and causes hypoxia, resulting in strong SD dependence on slice thickness. Our model also predicts that the velocity of SD propagation is not dependent on its initiation, but instead on tissue properties, including the amount of extracellular space and the total area of neuronal membrane, suggesting faster SD following ischemic stroke or traumatic brain injury.

## Introduction

Spreading depolarization (SD) is a slow-moving (1.7–9.2 mm/min), long-lasting (minutes) wave of neuronal depolarization accompanied by a breakdown in homeostatic maintenance of intracellular and extracellular ion concentrations, and is associated with reduced neuronal activity (spreading depression; Dreier, 2011; Woitzik et al., 2013; Cozzolino et al., 2018; Newton et al., 2018). SD has been observed in a number of species, can be elicited experimentally both *in vivo* and in brain slices, and has been implicated in several neurologic conditions, including ischemia, migraine, traumatic brain injury (TBI), and epilepsy (Cozzolino et al., 2018). SD is difficult to detect in humans noninvasively (Drenckhahn et al., 2012; Hartings et al., 2014; Zandt et al., 2015; Hofmeijer et al., 2018), making it important to study SD in experimental preparations and computer simulation to better understand its role in human disease, and possible treatments.

SD has been studied in brain slices from a wide range of species and brain regions, including neocortex, hippocampus, brainstem, and retina (Balestrino et al., 1988; Aitken et al., 1998; Müller and Somjen, 1998; Martins-Ferreira et al., 2000; Devin Brisson et al., 2013; Andrew, 2016; Hrabe and Hrabetova, 2019). It can be triggered experimentally by various means, including electrical stimulation, mechanical insult, and K^+^ and ouabain application (Leao, 1944; Bures et al., 1974; Balestrino, 1995; Aitken et al., 1998; Joshi and Andrew, 2001). SD can be facilitated by applying propionate to the slice (Tao et al., 2002; Hrabe and Hrabetova, 2019).

SD is intimately related with hypoxia both in slice and *in vivo*. Ischemia plays a complex role in ischemic diseases: hypoxia can initiate SD, which then can contribute to the extent of the ischemic penumbra (Nedergaard, 1988); and SD can itself trigger ischemia. The scintillating scotomata of classical migraine is believed to be an SD wave and increases the risk of stroke (Øie et al., 2020); complicated migraine is caused by more severe ischemia, which produces more pronounced and long-lasting deficits (Santos et al., 2012). In *in vivo* experiments, SD also led to hypoxia (Takano et al., 2007; Piilgaard and Lauritzen, 2009). To begin examining this complexity in our simulations, we identified three types of hypoxia related to SD, comparable to different slice experiments: (1) we induced hypoxia to in turn induce depolarization, a phenomenon that has been called “hypoxic SD-like depolarization” (HSD; Balestrino et al., 1988; Aitken et al., 1991); (2) we used the “classical” SD initiation protocol (adding a K^+^ bolus to a slice) under hypoxic conditions, but before HSD had initiated, to compare to SD with an oxygenated bath; and (3) we looked at how SD induced hypoxia as perfusion from the bath failed to keep up with the metabolic demand of overworked Na^+^/K^+^ pumps.

**Figure 1.**
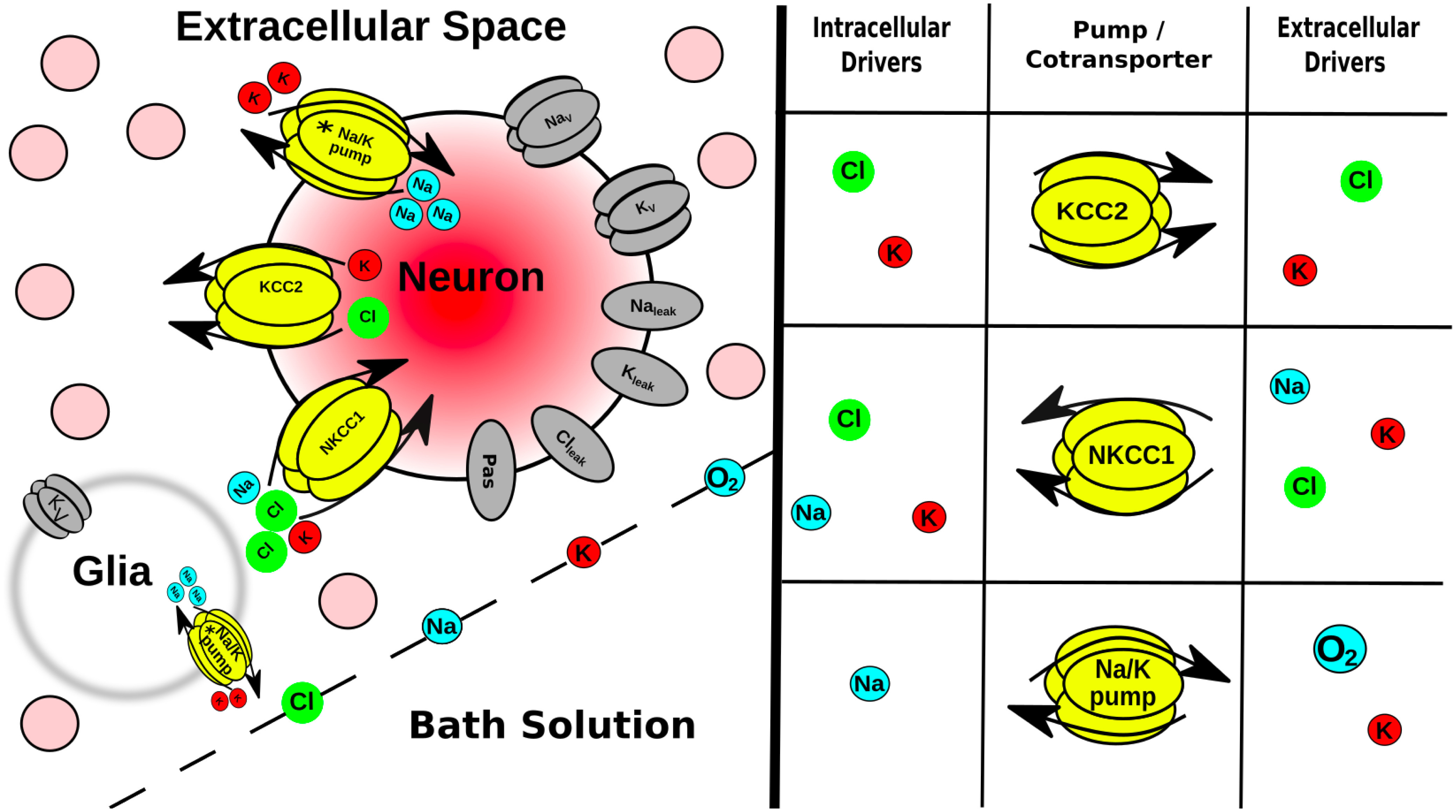
Multiscale model expanded. Tissue scale: a few of the 36 ⋅ 10^3^ neurons (pink circles) embedded in the ECS of a brain slice submerged in a bath solution where ion and O_2_ concentrations were held constant. Glia are not explicitly modeled, but instead were represented as a field of sinks in every ECS voxel. Cell scale: each neuron had ion channels, 2 coexchangers; Na^+^/K^+^ pump (asterisk indicates ATP/O_2_ dependence). Ions were well mixed within each neuron (no intracellular diffusion). Protein scale: table (right) indicates species that control the activity of the intrinsic mechanisms in neurons and in glial field. Ion scale: ions diffused between ECS voxels by Fick’s law using diffusion coefficients in Table 1.

In this article, we used multiscale computational modeling of SD to relate the microscopic levels of ion and O_2_ diffusion, channels, and pumps to the neuronal level of cell spiking up to the macroscopic level of tissue activation patterns (Fig. 1). Our baseline model was composed of 36,000 biophysically detailed point-neurons in an extracellular space (ECS) of a square slice (1 mm sides, 400 *μ*m thick) with O_2_ perfusion and ion flux with a surrounding bath where relevant concentrations are held constant at their baseline values. We simulated SD in both perfused and hypoxic slices. Our model showed that SD speed was augmented by propionate and hypoxia and suggested that changing the ECS was the principle mechanism through which they influence SD. We predicted that SD speed changes with slice thickness because of core hypoxia and increases with the total neuronal surface area in the tissue. SD speeds in all conditions were enhanced by hypoxia. We also predicted that increasing the size of the ECS relative to the tissue will inhibit SD. Finally, we identified a depth-dependent relationship with greater SD propagation through the core of the slice compared with the periphery.

**Table 1:** Diffusion coefficients and baseline concentrations for ions and O_2_ in perfused slice (Samson et al., 2003**;** Haynes, 2014**;** Wei et al., 2014**)**

Species	D (⋅10^–5^ cm^2^/s)	Intracellular concentration (mm)	ECS concentration (mm)
K^+^	2.62	140.0	3.5
Na^+^	1.78	18.0	144.0
Cl^–^	2.10	6.0	130.0
O_2_	3.30	0.1	0.1

## Materials and Methods

We developed a tissue-scale model of SD in slices by extending the framework developed by Wei et al. (2014) from a single cell in its local microenvironment to 36,000 cells (baseline) embedded in an ECS. We used the NEURON simulator and its extracellular reaction-diffusion framework (RxD-ECS) to simulate the electrophysiology of those neurons; the exchange of ions between them and the ECS; the diffusion of ions and O_2_ through the slice; and the exchange of ions and O_2_ between the slice and the bath solution in which it was submerged (Newton et al., 2018). Our model is not specific to any particular brain area, as we aimed to reproduce general properties applicable to different brain regions and to different pathophysiologies.

### Model

Model neurons were all point-neurons (one compartment), which included voltage-gated K^+^ and Na^+^ channels; Na^+^, K^+^, and Cl^–^ leak channels; KCC2 (K^+^-Cl^–^; Payne et al., 2003) and NKCC1 (Na^+^-K^+^-Cl^–^) cotransporters; and Na^+^/K^+^ pumps (Fig. 1), ported from the study by Wei et al. (2014). In order for cells to balance a steady-state resting membrane potential in a slice with dynamic ion and O_2_ concentrations, we added a passive electrical conductance with reversal potential (*E*_rev_ = –70 mV) and conductance (*g *=* *0.1 mS/cm^2^). Neurons were closed-ended cylinders (no flux from the ends). The Na^+^/K^+^ pump activity was dependent on the local concentration of O_2_ in the slice, rather than ATP, and Na^+^/K^+^ pumps were the only consumers of O_2_ in the model (Wei et al., 2014). ATP reserve in rat cells is estimated to be ∼2.6 mm (Veech et al., 1979). A single-cell simulation using a Michaelis–Menten approximation for ATP dependence on O_2_ (Noske et al., 2010) demonstrated that [K^+^]_ECS_ = 15 mm increased pump activity by ∼1.8×, which would reduce reserves to <1% within 2 s under hypoxic conditions.

To explore the effects of surface area to volume ratio (S/V), we used RxD-ECS to independently define a neural surface entirely separated from its volume—hence not following the overall geometry of the structure. This is possible since we used the concept of fractional volumes, rather than providing actual volume-occupying structures (McDougal et al., 2013). Neuronal volume fraction (*β*_nrn_) is defined as the ratio of total neuronal volume (Vol_nrn_) to total tissue volume: $\beta_{\text{nrn}}=\frac{{\text{Vol}}_{\text{nrn}}}{{\text{Vol}}_{\text{ tissue}}}$ (compare with α_ECS_, which is $\frac{{\text{Vol}}_{\text{ECS}}}{{\text{Vol}}_{\text{ tissue}}}$; Rice and Russo-Menna, 1997). Given a chosen tissue volume, *β*_nrn,_ and a total number of neurons, *N*_nrn_ (based on cell density times Vol_tissue_), the volume of a single neuron, vol_nrn_, is as follows:

$${\text{vol}}_{\text{nrn}}=\frac{\beta_{\text{nrn}}\cdot {\text{Vol}}_{\text{ tissue}}}{N_{\text{nrn}}},$$

(note the case used: vol_nrn_ for single cell; Vol_nrn_ for cumulative neuronal volume). In the NEURON simulator, neural compartments are cylinders, omitting the ends. In the present case, our point-neurons are each a single cylinder, defined by length *L* and diameter *d*. Setting *L *=* d* for simplicity, the surface area is defined as $S_{\text{nrn}}=\pi \cdot d^{2}$. The associated volume, calculated for this cylinder, was not used. We therefore used the FractionalVolume class of RxD-ECS to scale the volume of a cell with the desired S/V to vol_nrn_, while the cell retains its original *S*_nrn_ (McDougal et al., 2013).

To establish a biologically realistic range for S/V, we analyzed morphologic reconstructions of neurons from neuromorpho.org. We used results for cells with intact soma and dendritic reconstructions in three dimensions from animals >2 weeks of age. In rat neocortex, the average S/V was 3.4 ± 1.2 *μ*m^– 1^ for both pyramidal cells (*n* = 96) and interneurons (*n* = 108; Vetter et al., 2001; Larkum et al., 2004; Radman et al., 2009; Meyer et al., 2010; Boudewijns et al., 2013; Kubota, 2014). Higher S/V was grossly associated with larger dendritic trees; S/V scales inversely with diameter in cylindrical structures (excluding ends), as follows: S/V = $\frac{4}{d}$. For our baseline simulations, we used S/V = 3.0 *μ*m^–1^; β_nrn_ = 0.24; neuronal density = 90 ⋅ 10^3^ neurons/mm^3^, typical of neocortex (Rice and Russo-Menna, 1997; Keller et al., 2018).

Simulated slices were 1 × 1 mm and ranged in thickness from 100 to 800 μm with 45 ⋅10^3^–120 ⋅10^3^ neurons/mm^3^. The baseline simulation was a 400 *μ*m slice with 90 ⋅10^3^ neurons/mm^3^ (36 ⋅10^3^ neurons in total). Neurons were situated randomly throughout ECS with diffusion of Na ${}_{\text{ECS}}^{\text{+}}$, K ${}_{\text{ECS}}^{\text{+}}$, Cl ${}_{\text{ECS}}^{{}^{–}}$, and O_2_, with diffusion coefficients (D) given in Table 1. Extracellular volume fraction (*α*_ECS_: the ratio of extracellular space volume to total tissue volume) and tortuosity (*λ*_ECS_: hindrance to diffusion in tissue compared with free medium) were the same for all ions. This results in a lower effective diffusion coefficient (D*) for ions but not O_2_, which diffused through the slice unhindered. Diffusion through the ECS was calculated with a voxel size of 25 × 25 × 25 *μ*m. Simulated slices were submerged in simulated bath solution where ion and O_2_ concentrations were equivalent to those estimated for ECS (Table 1; Cressman et al., 2009; Wei et al., 2014) with Dirichlet boundary conditions.

In the perfused slice, [O_2_] = 0.1 mm [corresponding to a bath solution aerated with 95% O_2_:5% CO_2_ (Wei et al., 2014), *α*_ECS_ = 0.2, and *λ*_ECS_ = 1.6 (Syková and Nicholson, 2008)]. We modeled the effects of propionate, which “primed” the tissue for SD, by reducing *α*_ECS_ to 0.12 and total Cl^–^ content in the slice by 50%, but keeping [O_2_] = 0.1 mm and *λ*_ECS_ = 1.6 (Hrabe and Hrabetova, 2019). In both cases, SD was initiated by elevating initial [K^+^]_ECS_ within a spherical bolus at the center of the slice at *t* = 0. Baseline simulations were run with K^+^ bolus with radius = 100 *μ*m; [K^+^]_ECS_ = 70 mm. Hypoxia can also induce a depolarization, termed by some HSD, without applying a K^+^ bolus (Pérez-Pinzón et al., 1995; Aitken et al., 1998). Experimentally, switching the gas to 95% N_2_:5% CO_2_ leads to HSD within minutes, but immediately preceding the depolarization, Pérez-Pinzón et al. (1995) identified a period where *α*_ECS_ is reduced and *λ*_ECS_ is increased, which they termed the “preanoxic depolarization phase.” To simulate these conditions, we reduced *α*_ECS_ to 0.13, increased *λ*_ECS_ to 1.8 (Pérez-Pinzón et al., 1995), reduced [O_2_] in the slice to 0.01 mm, and increased K^+^ to 15 mm in a 100-*μ*m-radius sphere in the center of the slice, providing a nidus for depolarization initiation. In studies by both Pérez-Pinzón et al. (1995) and Aitken et al. (1998), the slice was reperfused immediately after detecting the depolarization, so we set the boundary conditions such that [O_2_] in the bath was 0.1 mm (full bath oxygenation). We also simulated a scenario in which a 70 mm bolus of K^+^ was applied to the slice during the preanoxic depolarization phase but before the HSD occurs, and the slice was not reperfused to make direct comparisons between SD in a hypoxic slice and SD in a perfused slice. This could be accomplished experimentally by continuously monitoring ECS properties to determine when to apply the K^+^ bolus.

Studies have shown that *α*_ECS_ changes dynamically over the course of SD (Mazel et al., 2002; Hrabe and Hrabetova, 2019). Since the biophysics of ECS changes during SD have not been elucidated at the timescale of our simulations, we incorporated these changes phenomenologically for a subset of simulations, reducing *α*_ECS_ to 0.05–0.1 during passage of the SD (Mazel et al., 2002) in each ECS voxel, as follows:

$$\frac{d\;\alpha_{\text{ECS}}}{dt}=(5\cdot 10^{-7})\cdot ({\left[K^{+}\right]}_{\text{ECS}}-3.5)\cdot ({\left[K^{+}\right]}_{\text{ECS}}-70.0)\cdot \alpha_{\text{ECS}}.$$

This model of dynamic *α*_ECS_ only accounts for the drop in *α*_ECS_ during SD, not during its recovery after SD, which occurs on the timescale of minutes (Mazel et al., 2002; Hrabe and Hrabetova, 2019).

Glia were modeled by a background voltage-gated K^+^ current and Na^+^/K^+^ pump in each ECS voxel (Cressman et al., 2009; Øyehaug et al., 2012; Wei et al., 2014), rather than as individual cells.

### Simulations

SD was initiated with K^+^ bolus with radius = 100 *μ*m; [K^+^]_ECS_ = 70 mm, unless noted otherwise. To follow the position of SD over time, we tracked the position of the K^+^ wavefront, defined as the farthest location where [K^+^]_ECS_ exceeded 15 mm, and the positions of spiking neurons. Propagation speed was indicated as 0 if the K^+^ wave did not propagate. Most simulations ran for 10 s, which was sufficient for SD to propagate throughout the entire slice. Because of computational limitations, we restricted our simulations to 10 s, which meant that we could not continue to the termination phase of SD, which would require considerably greater temporal and spatial scales.

### Code accessibility

All codes for simulation, data analysis, and visualization described in this article were written in Python/NEURON and are freely available on GitHub (Extended Data 1). In the course of this study, we ran >600 simulations covering a range of slice sizes, cell densities, and durations on a number of different architectures. Simulating a 1 × 1 × 400 *μ*m slice with a cell density of 90,000 neurons/mm^3^ (36 ⋅10^3^ neurons) for 10 s of simulation time took ∼12.5 h on a parallel Linux system using 48 nodes on a 2.40 GHz Intel Xeon E5-4610 CPU. Incorporating dynamic *α*_ECS_ into the same model on the same machine increased simulation time to ∼18 h. Simulations were run using Neuroscience Gateway (Sivagnanam et al., 2013), Google Cloud Platform, and the on-site high-performance computer at SUNY Downstate Health Sciences University. Smaller versions of the model can be run on a personal computer.

10.1523/ENEURO.0082-22.2022.ed1 Extended Data 1 Simulation code: our tissue-scale model of SD in brain slices is available on GitHub. We used the NEURON simulator reaction-diffusion framework to implement embed thousands of neurons (based on the model from Wei et al., 2014) in the extracellular space of a brain slice, which is itself embedded in an bath solution. We initiated SD in the slice by elevating extracellular K^+^ in a spherical region at the center of the slice. The effects of hypoxia and propionate on the slice were modeled by appropriate changes to the volume fraction and tortuosity of the extracellular space and oxygen/chloride concentrations. Users need to install NEURON, and we recommend using MPI to parallelize simulations. Download Extended Data 1, ZIP file.

### Code availability

The model is publicly available on GitHub and ModelDB. The access code for ModelDB is *Airy1870*.

## Results

**Figure 2.**
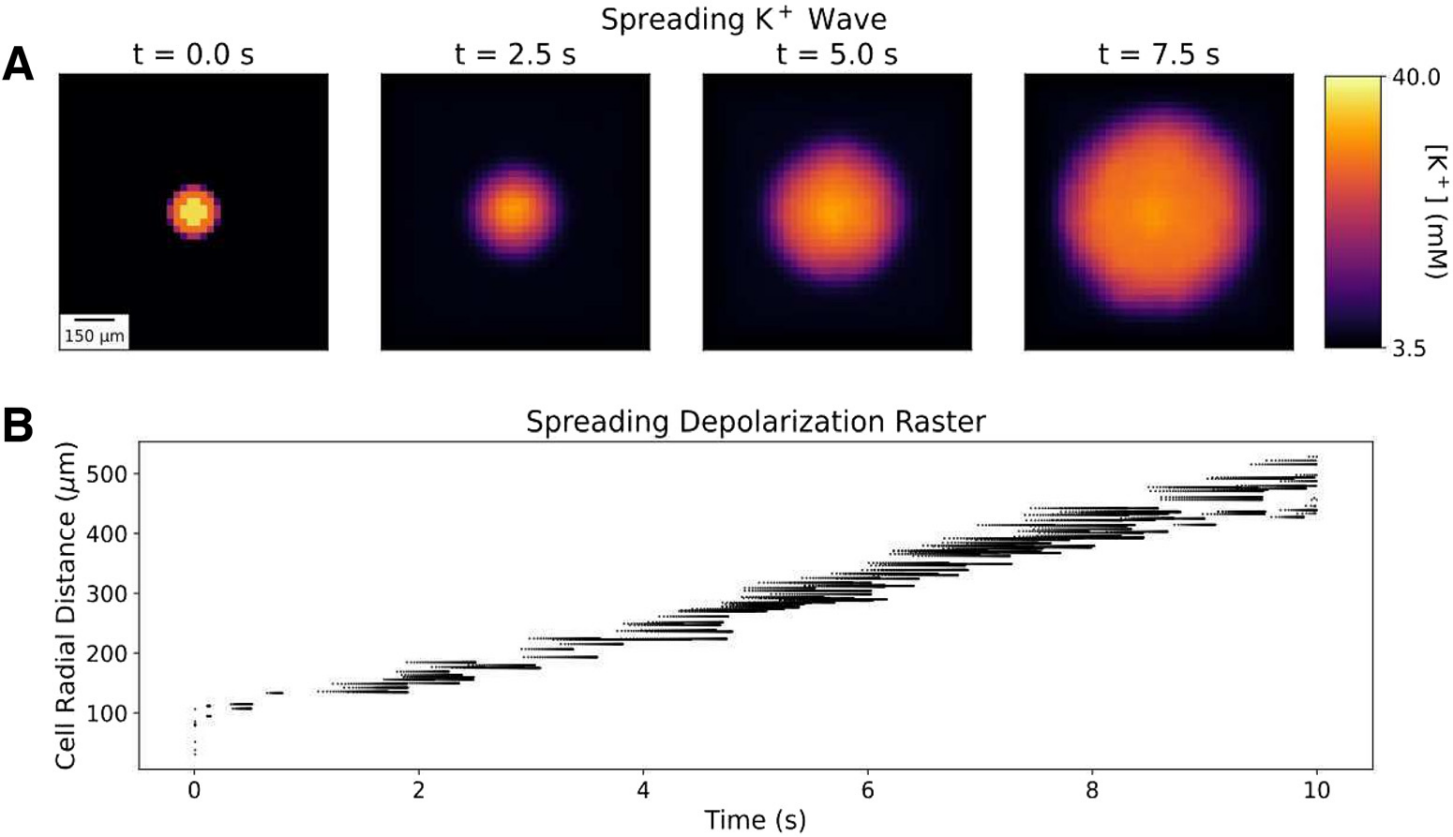
A small bolus of applied K^+^ initiates spreading K^+^ and depolarization waves in perfused slice. ***A***, [K^+^]_ECS_ averaged across slice depth (400 *μ*m) at 4 time points during SD. ***B***, Spike raster plot of 250 randomly selected neurons (of 36 ⋅10^3^) during SD. Cells are ordered on *y*-axis by their radial distance from the center of the K^+^ bolus. Blank area under spikes represents region of spreading depression. Baseline values: [O_2_] = 0.1 mm; *α*_ECS_ = 0.2; *λ*_ECS_ = 1.6; [Cl^–^]_ECS_ = 130.0 mm; [Cl^–^]*_i_* = 6.0 mm.

In our model of an O_2_-perfused slice, a small bolus of elevated K^+^ (70 mm, 100 *μ*m radius) initiated a propagating K^+^ wave with associated SD producing neuronal spiking (Fig. 2). The K^+^ wave traveled radially outward from the bolus in three dimensions toward the edges of the slice at 2.3 mm/min, comparable to optical and electrophysiological measurements of SD propagation velocity in brain slices (Aitken et al., 1998; Joshi and Andrew, 2001; Hrabe and Hrabetova, 2019). Within the K^+^ bolus, most cells fired a single spike and went immediately into depolarization block. Outside the K^+^ bolus, cells fired a 200–900 ms burst of action potentials as [K^+^]_ECS_ increased around them. During the course of the SD-associated burst, instantaneous firing rates increased to as high as 250 Hz with decreasing spike heights during the burst, comparable to experimental observations (Devin Brisson et al., 2013; Lemaire et al., 2021). Cells then remained in depolarization block for the remainder of the 10 s measured (see Materials and Methods for computational limitations; Devin Brisson et al., 2013; Andrew, 2016). Spreading depolarization, seen intracellularly, produced Na^+^ channel inactivation and prevented further spiking. The absence of spiking would be seen extracellularly as spreading depression—a silent region in the slice. The K^+^ wave and SD were coincident in time and space, with spreading depression following closely behind; we primarily followed the K^+^ wave since this was easiest to localize across conditions.

**Figure 3.**
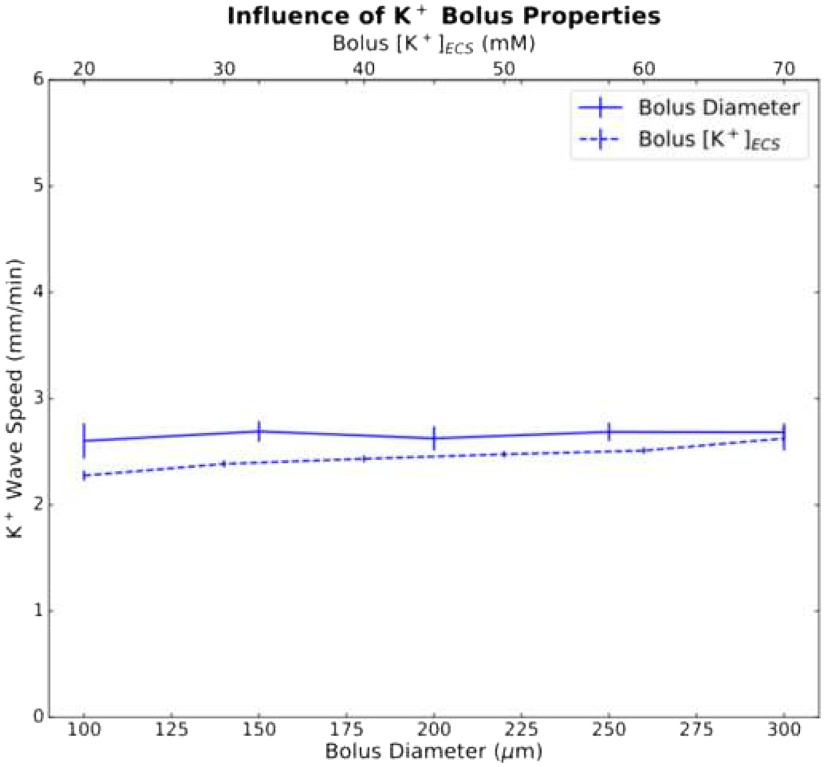
Diameter (bottom, *x*-axis) and concentration (top, *x*-axis) of the K^+^ bolus had minor effects on K^+^ wave speed. Mean and SDs (*n* =* *5) for K^+^ wave speed versus bolus diameter (solid line; bolus [K^+^]_ECS_ = 70 mm; 5 random cell position initializations) and versus bolus concentration (dashed line; diameter, 200 *μ*m).

SD in perfused slices was an all-or-none process; it could only be initiated above a certain threshold measured either in concentration—[K^+^]_ECS_ ≥ 20 mm (bolus diameter, 200 *μ*m) or [K^+^]_ECS_ = 70 mm (bolus diameter ≥ 100 *μ*m; Fig. 3). Beyond these thresholds, different K^+^ bolus concentrations and diameters had only a minimal effect on wave speed.

**Figure 4.**
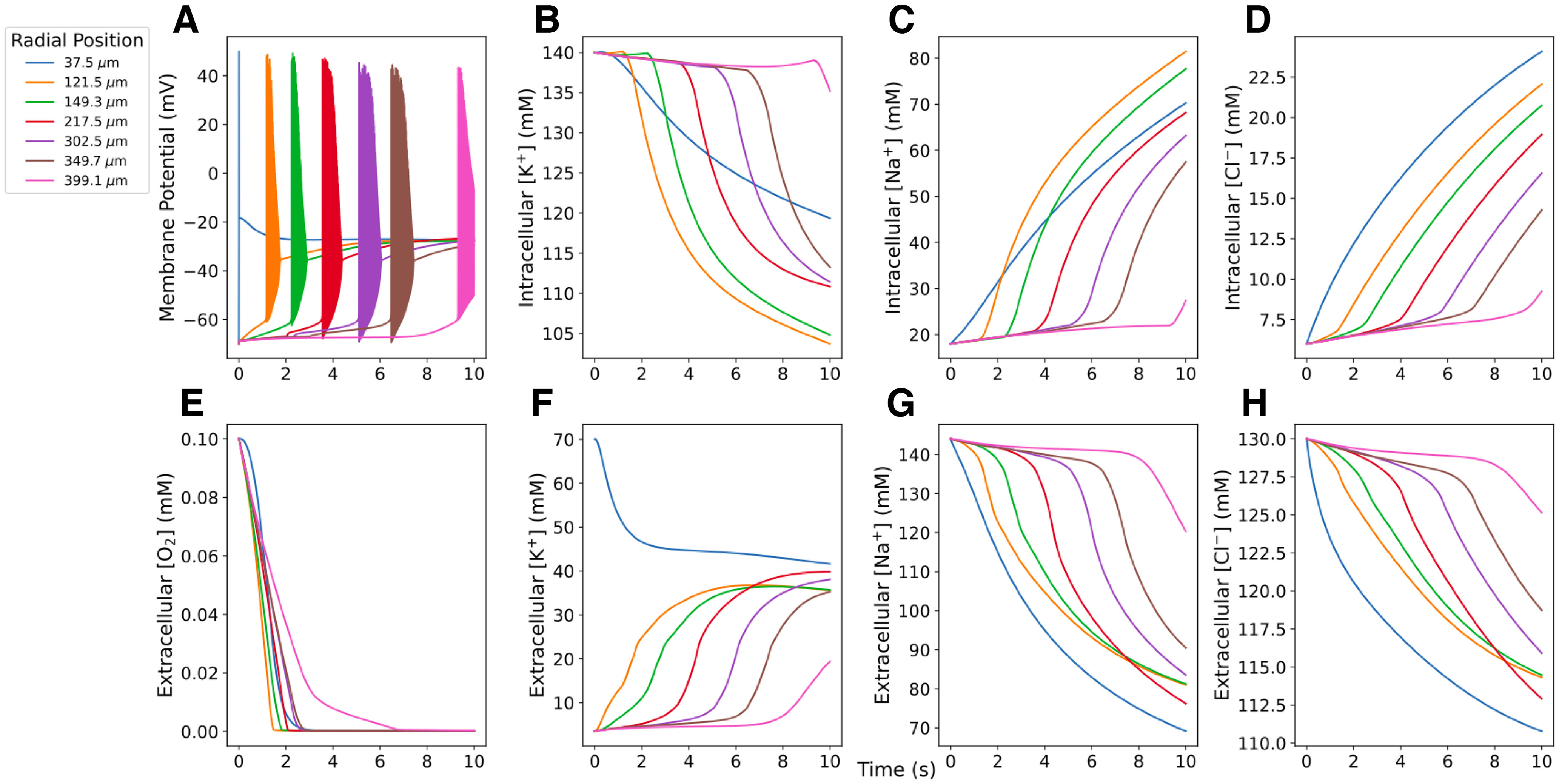
Concentrations at 7 radial locations measured during SD in perfused 400 *μ*m slice. ***A***, Cell within the K^+^ bolus (37.5 *μ*m) produced a single spike; cells farther out fired a burst. Cells remained in depolarization block for the remainder of the simulation (10 s). ***B–D***, Intracellular ion concentrations. ***E–H***, Extracellular O_2_ and ion concentrations in neighboring ECS voxels. Movie 1 shows extracellular ion and O_2_ concentrations across the slice, as well as neuronal spiking (white dots) from 250 neurons during the course of SD (most easily seen with slowed playback).

Underpinning SD was a wave of pronounced imbalance of transmembrane ion concentrations (Fig. 4, Movie 1). Excess K^+^ is briefly eliminated from the ECS via neural and glial homeostatic mechanisms. Once the K^+^ wave arrived, however, the cells dumped large quantities of K^+^ into the ECS because of the burst and subsequent prolonged depolarization (note the rapid depletion of [K^+^]*_i_*; Fig. 4*B*). The Na^+^/K^+^ pump activity that contributed to K^+^ elimination from the ECS in the core of the slice created a high demand for O_2_, exceeding the rate at which it could diffuse in from the bath. This resulted in much of the tissue becoming hypoxic before the arrival of the K^+^ wave (Fig. 4, compare *E*, rapid falloff of O_2_, *F*, much slower rise of extracellular K^+^). The rapid spread of O_2_ deficit explains the total pump failure at intermediate locations in the slice. There were small upward deflections in the first three traces in Figure 4*B* reflecting homeostatic inward pumping. There was no upward deflection in the other three traces—O_2_ has disappeared before the K^+^ wave arrives. The preservation of pumping in the final, most peripheral, trace of Figure 4*B* is because of this measurement being at the edge of the slice, neighboring the O_2_ source of the bath. In reality, neuronal ATP reserves will maintain pumping for a limited duration in the absence of O_2_, but reserves will quickly run out because of the high metabolic demand imposed on the pumps by large shifts in K^+^ and Na^+^ concentrations. Once [K^+^]_ECS_ reached ∼14 mm, cellular homeostatic mechanisms totally broke down. Changes in intracellular and extracellular Na^+^ concentrations were actually larger than the shifts in K^+^ accompanying SD, and a wave of extracellular Na^+^ deficit traveled along with the K^+^ wave (Fig. 4*C*,*G*). Shifts in Cl^–^ concentrations proceeded through the slice slightly more slowly and were less pronounced (Fig. 4*D*,*H*).

Movie 1. Extracellular ion and O_2_ concentrations across the slice averaged over depth, as well as neuronal spiking (white dots) from 250 neurons during the course of SD in a perfused slice. The spread of spiking and the K^+^ wave can be seen in real time. We recommend downloading the file and using slower playback to visualize the spread of hypoxia. 10.1523/ENEURO.0082-22.2022.video.1

**Figure 5.**
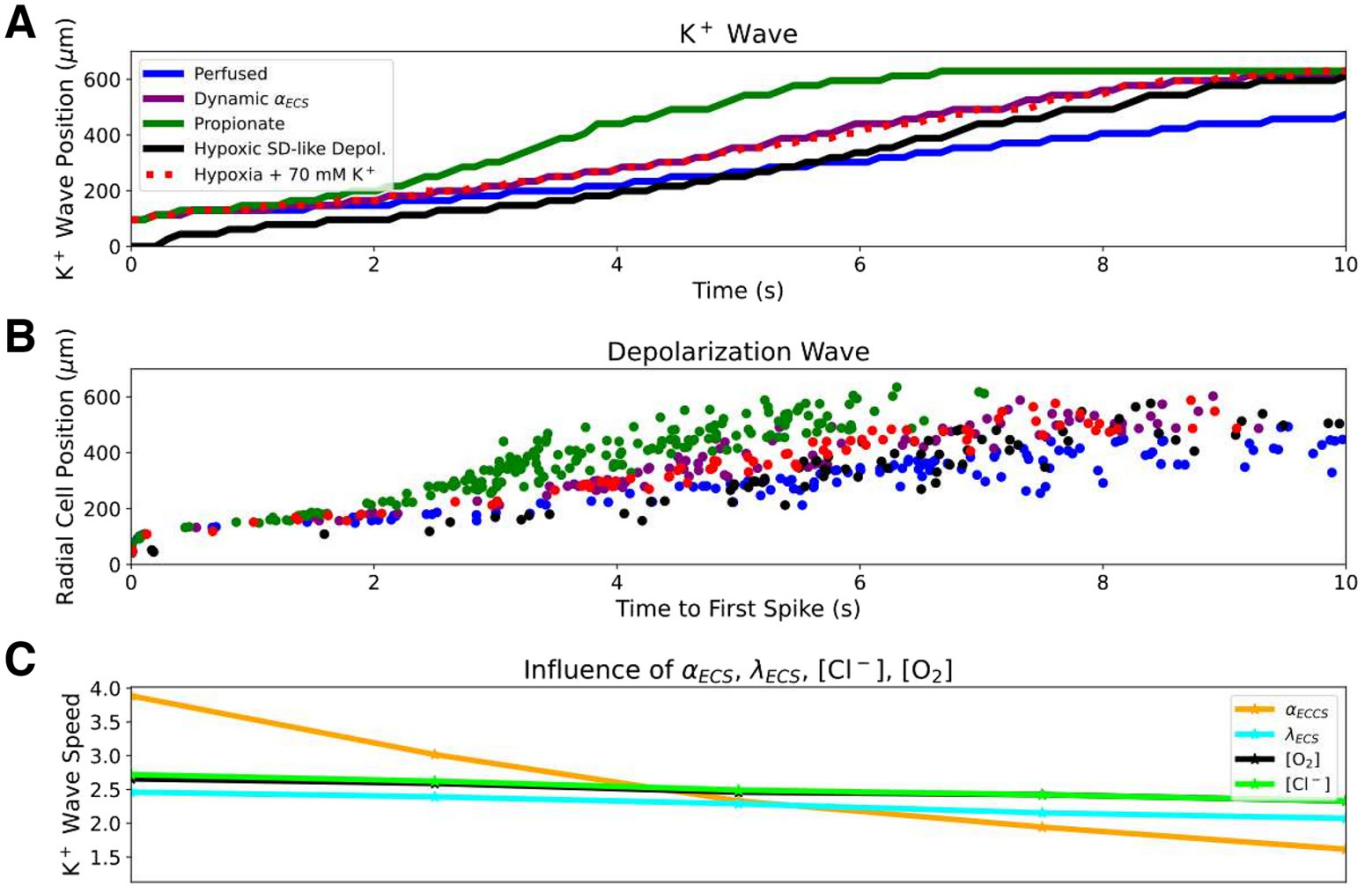
Hypoxia, propionate, and dynamic ECS increased SD speed principally through *α*_ECS_ reduction. ***A***, Radial K^+^ wave position over time during SD in perfused (Movie 1), hypoxic, HSD (Movie 2), propionate conditions, and with dynamic changes in ${\text{α}}_{\text{ECS}}$. Hypoxia, propionate, and dynamic changes in *α*_ECS_ facilitated propagation. ***B***, Radial position of SD wave represented by time to first spike in 126 selected cells at different distances from center. ***C***, K^+^ wave speeds with individual parameter changes (Fig. 2). *α*_ECS_ had the greatest impact on SD speed over a physiologically plausible range (*x*-axis ranges: [O_2_] = 0.01–0.1 mm; *α*_ECS_ = 0.07–0.42; *λ*_ECS_ = 1.4–2.0; [Cl^–^]_ECS_:[Cl^–^]*_I_* = 3.0:65.0–6.0:130.0 mm).

SD was facilitated by incorporating the effects of hypoxia or propionate treatment on the slice. HSD (caused by hypoxia, rather than introducing a K^+^ bolus) propagated similarly to SD in a perfused slice, as shown experimentally (Aitken et al., 1998; Fig. 5). Pérez-Pinzón et al. (1995) identified a period immediately preceding the HSD they termed a preanoxic depolarization phase, with reduced *α*_ECS_ and increased *λ*_ECS_. We modeled HSD by incorporating these hypoxia effects, using a small region of slightly elevated K^+^ (100 *μ*m radius, 15 mm) to provide a nidus for HSD initiation. The spatiotemporal distribution of neuronal spiking was similar to that of the control (perfused) slice, as seen experimentally (Fig. 5*B*, Movie 2). However, the K^+^ wave during HSD was faster (3.7 mm/min) than during SD in the perfused slice (2.3 mm/min; Fig. 5*A*). We also simulated standard K^+^-initiated SD in hypoxic slices by applying a 70 mm K^+^ bolus (as with SD in the perfused slice) to a slice during this preanoxic depolarization phase (Pérez-Pinzón et al., 1995), resulting in K^+^ wave speed of 3.4 mm/min. Simulating propionate application (decreased *α*_ECS_ = 0.12; halving [Cl^–^]*_i_* and [Cl^–^]_ECS_; Tao et al., 2002; Hrabe and Hrabetova, 2019), increased K^+^ wave speed to 4.8 mm/min (Fig. 5*A*). Comparable speedups were also observed in the depolarization waves (Fig. 5*B*). Since these manipulations included combined changes to [O_2_], Cl^–^, *α*_ECS_, and/or *λ*_ECS_, we investigated their individual contributions over the relevant ranges. Since both hypoxia and propionate decrease *α*_ECS_, we also tested increasing *α*_ECS_ to as high as 0.42, which has been observed when making the ECS artificially hypertonic (Kume-Kick et al., 2002). *α*_ECS_ had the largest influence on propagation, changing K^+^ wave speed by >2 mm/min over the range tested, while K^+^ wave speed changed by <0.5 mm/min for the ranges of [O_2_], *λ*_ECS_, and [Cl^–^] values tested (Fig. 5*C*).

Movie 2. Extracellular ion and O_2_ concentrations across the slice averaged over depth, as well as neuronal spiking (white dots) from 250 neurons during the course of hypoxic SD-like depolarization. The spread of spiking and the K^+^ wave can be seen in real time. We recommend downloading the file and using slower playback to visualize the delay between initiating the simulation and the spread of the K^+^ wave. 10.1523/ENEURO.0082-22.2022.video.2

Experimental studies have demonstrated dynamic changes in *α*_ECS_ occurring during SD, with *α*_ECS_ dropping to as low as 0.05 at the peak of the depolarization (Mazel et al., 2002; Hrabe and Hrabetova, 2019). Given the strong influence of constant *α*_ECS_ on SD propagation (Fig. 5*C*, orange line), we also explored the influence of dynamic *α*_ECS_ (Fig. 5*A*, purple line). Dynamically decreasing *α*_ECS_ was modeled as a local function of increasing [K^+^]_ECS_ (see Materials and Methods), such that *α*_ECS_ dropped to 0.06 in the wake of SD, within the experimentally observed range of 0.05–0.1 (Mazel et al., 2002). Incorporating a dynamic ECS increased the speed of SD propagation in perfused slice from 2.3 to 3.3 mm/min. The stereotyped characteristics of neuronal firing patterns during the depolarization wave remained unchanged by dynamic changes in *α*_ECS_.

**Figure 6.**
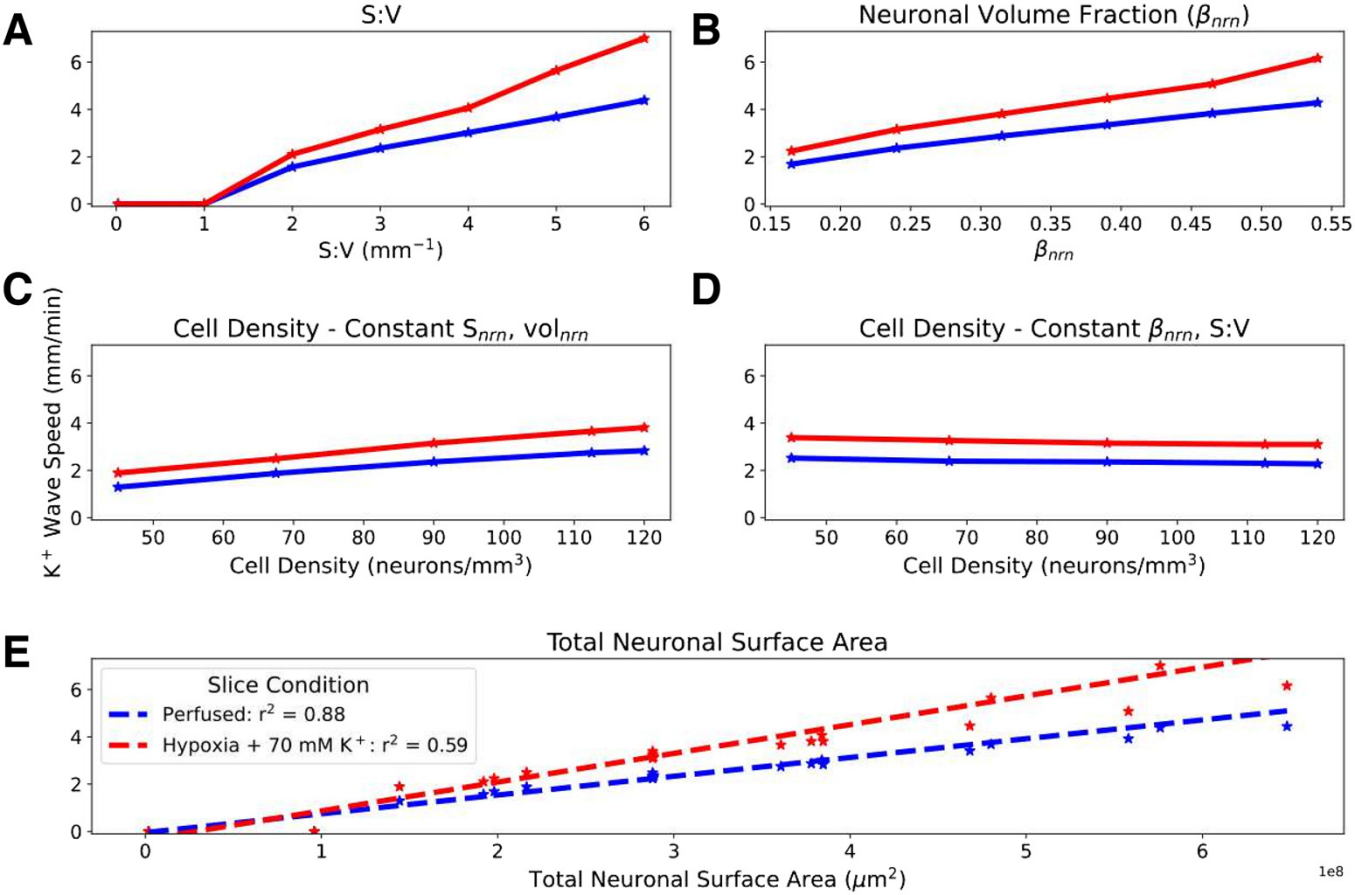
K^+^ wave propagation speed proportional to total neuronal surface area in slice. SD initiated in perfused and hypoxic slices (no reperfusion) by introducing a 100-*μ*m-radius central 70 mm spherical K^+^ bolus. ***A–E***, Effects of varying S/V of each cell while maintaining a constant *β*_nrn_ (***A***); varying *β*_nrn_ while keeping S/V constant, thus allowing S_nrn_ to vary (***B***); varying cell density while keeping constant S_nrn_ and vol_nrn_, thus allowing *β*_nrn_ to vary (***C***); and varying cell density while keeping *β*_nrn_ and S/V constant, thus allowing S_nrn_ and vol_nrn_ to vary (***D***). ***E***, Pooled results: K^+^ wave speed increased linearly with total neuronal surface area in both perfused and hypoxic slices. Hypoxia increased wave speed across conditions (0 speed indicates no SD).

Different brain areas have different cell densities, and their neurons have different morphologic characteristics. We manipulated our generic model so as to explore the following three properties of neural tissue organization and shape: neuronal S/V; the fraction of tissue volume occupied by neurons (*β*_nrn_); and cell density (number of neurons per mm^3^; Fig. 6). Neuronal S/V varies across cell types, brain regions, and species. Examination of representative morphologies showed that S/V values are generally in a range of 2–10 *μ*m^– 1^ (see Materials and Methods), with neocortical principal cell S/V of 3.4 ± 1.2 *μ*m^– 1^ (*n* = 96), which is significantly greater than the brainstem principal cell S/V of 2.2 ± 1.2 *μ*m^– 1^ (*n* = 74; *p* <0.001, Mann–Whitney *U* test; Núñez-Abades et al., 1994; Vetter et al., 2001; Larkum et al., 2004; Radman et al., 2009; Meyer et al., 2010; Ros et al., 2010; Boudewijns et al., 2013; Raslan et al., 2014; Williams et al., 2019). Neuronal volume fraction *β*_nrn_ may differ with different brain areas and will differ under the pathologic condition of cytotoxic edema. Cell density varies across different neural areas.

Realistic (>1 *μ*m^– 1^) S/V was necessary for initiating SD (Fig. 6*A*)—SD could not be initiated using the actual 3D geometry of single-cylinder point-neurons with diameter and height selected to produce a baseline *β*_nrn_ of 0.24 (S/V = 0.02 *μ*m^– 1^ with 90,000 neurons/mm^3^, perfused or hypoxic). Above this threshold, K^+^ wave speed increased with S/V. K^+^ wave speed also increased with increased *β*_nrn_ while keeping S/V and cell density (number of neurons per mm^3^) constant (Fig. 6*B*). Cell density effects were less marked, whether through keeping surface area and volume constant (Fig. 6*C*) or keeping *β*_nrn_ and S/V constant (Fig. 6*D*). In all cases, change with altered parameters was more pronounced in the hypoxic slice. Pooled together, we found a near linear relationship of K^+^ wave speed with total neuronal surface area.

**Figure 7.**
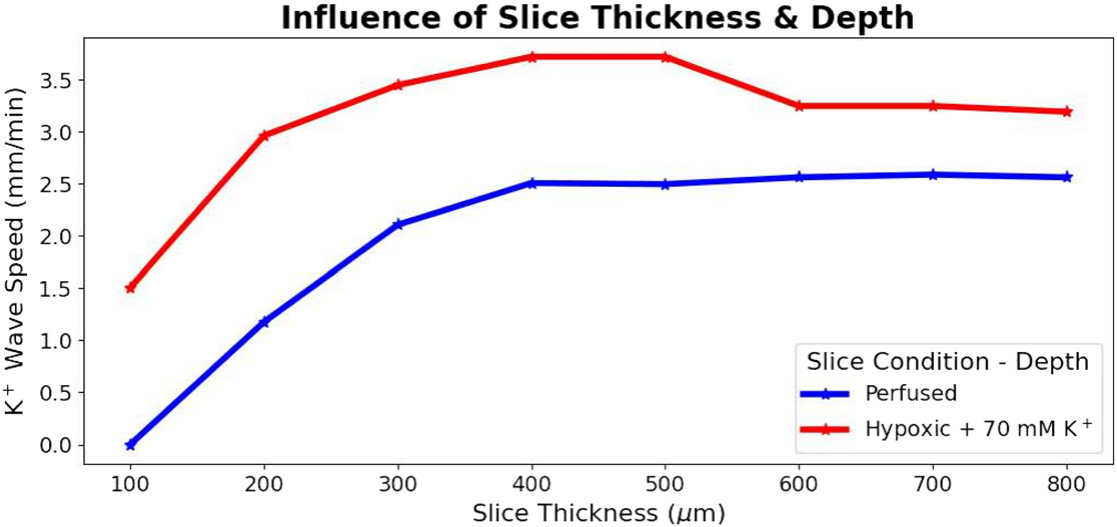
Slice thickness effects on SD propagation. K^+^ wave speed during SD in perfused and hypoxic slices of various thicknesses. SD could not be initiated in very thin (100 *μ*m) slices when perfused but could in hypoxic slices. For perfused slices, K^+^ wave speed increased with slice thickness between 200 and 400 *μ*m then saturated for slices of greater thickness. A similar pattern was observed in the hypoxic slices with consistently faster K^+^ wave speeds compared with in perfused slices.

Slice thickness (100–800 *μ*m) influenced SD by altering the ability of O_2_ to penetrate to the tissue core (Fig. 7). SD could not be initiated in the 100 *μ*m perfused slice—SD was not sustainable with full O_2_ availability, but it was initiated in an hypoxic slice of the same thickness. With increasing thickness, an increasingly hypoxic core (despite O_2_ perfusion of the bath) allowed K^+^ wave speed to increase from 1.2 to 2.1 mm/min over 200–400 *μ*m thickness (Fig. 7*A*). Above 400 *μ*m, there was no increased speed with increased thickness. Similar patterns were observed in hypoxic slices.

**Figure 8.**
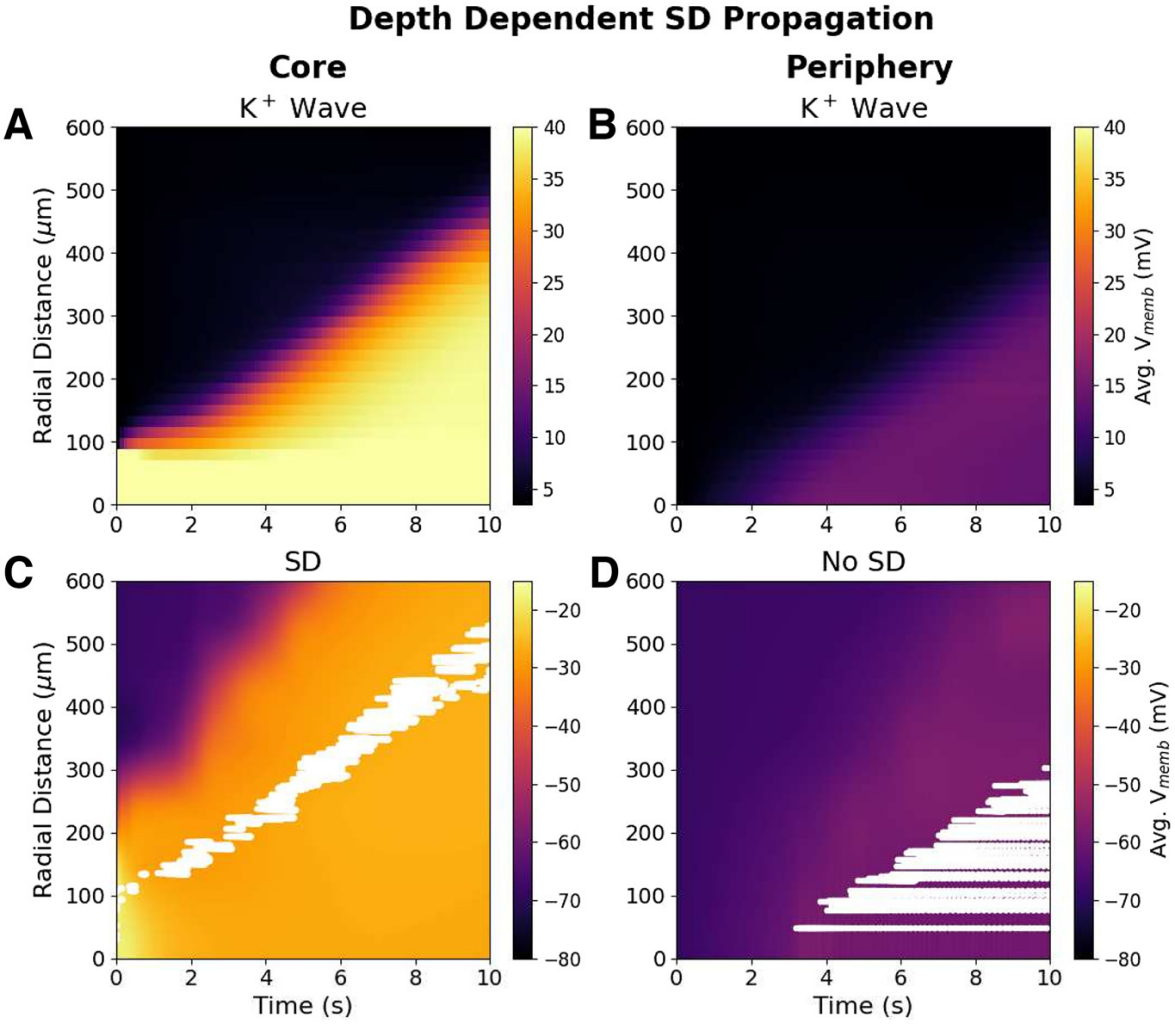
Depth dependence of SD propagation in 400-*μ*m-thick perfused slice. ***A***, Spread of K^+^ wave through slice core (±50 *μ*m from center). ***B***, Wave of mildly elevated K^+^ reached the periphery (within 50 *μ*m of bath) from the central bolus. ***C***, Spread of SD through the slice core. Voltages in slice core (color map) with spike rasters for 120-cell subset overlaid in white. Neurons in the core showed typical SD voltage dynamics (bursting followed by depolarization block). ***D***, Voltages and raster at periphery; cells show regular spiking patterns at 10–70 Hz (*V*_memb_ averaged across all cells in 25 × 25 × 100 *μ*m voxels at center or periphery).

We also observed depth-dependent differences in propagation of the SD within a slice (Fig. 8). A wave of high K^+^ propagated through the core of the slice (±50 *μ*m from the center; Fig. 8*A*), and neurons there exhibited the membrane dynamics of SD: a burst of spikes followed by depolarization block lasting the remainder of the simulation (Fig. 8*C*). The K^+^ wave reached the periphery of the slice (within 50 *μ*m of bath) but in lower concentration (Fig. 8*B*), and neurons there were resistant to SD because of the high availability of O_2_ from the bath (Fig. 8*D*). Instead, neurons in the periphery of the slice only underwent a modest depolarization and fired regularly at 10–70 Hz, without the bursting followed by depolarization block characteristic of SD.

## Discussion

Our model reproduced a number of the properties of SD observed in brain slices (Figs. 2, 4). Slice models that most resembled cortical gray matter (high cell density, high neuronal S/V) showed SD speeds and neuronal firing patterns comparable to *in vitro* optical and electrophysiological measurements (Aitken et al., 1998; Devin Brisson et al., 2013; Hrabe and Hrabetova, 2019; Lemaire et al., 2021). The all-or-none nature of SD initiation, as well as the observed bolus [K^+^] threshold of ∼20 mm, was also in agreement with experiments in brain slices (Andrew et al., 2017).

Our simulations identify a pathologic cycle whereby SD induces, and is also exacerbated by, hypoxia in slice. *In vivo*, SD-associated metabolic demand for O_2_ can exceed supply, resulting in tissue hypoxia (Takano et al., 2007; Piilgaard and Lauritzen, 2009). However, *in vivo* experiments showed hypoxia following SD, rather than preceding it as predicted by our *in vitro* simulations. *In vivo*, O_2_ is supplied by the vasculature, and increased neural activity during SD may lead to increased blood supply and tissue oxygenation (Balança et al., 2017), while *in vitro*, O_2_ is only supplied by the bath, which is effectively unchanging. While K^+^ was slowly spreading outward across the slice, O_2_ spread inward rapidly, following the gradient caused by O_2_ depletion from overworked pumps, but was quickly consumed (Fig. 4, compare *E*, rapid falloff of O_2_, *B*, much slower falloff of [K^+^]*_i_*). Depending on the distance to the bath and to the inciting bolus, intermediate locations in the slice suffered various degrees of pump demand and partial pump failure. The resistance to SD of neurons in the periphery of the slice compared with those in the core (Fig. 8*B*) was comparable to findings *in vivo*: tissue near capillaries resist SD, while tissue farther away is relatively hypoxic and prone to SD (Takano et al., 2007).

By comparing the effects of changing O_2_ availability, total Cl^–^ content, *α*_ECS_, and *λ*_ECS_ on SD in isolation, we determined that *α*_ECS_ influenced SD most strongly (Fig. 5*C*), accounting for most of the speedup observed in hypoxic and propionate-treated slices. Our results support the hypothesis that the main mechanism in the priming of propionate for SD is through reducing *α*_ECS_ (Hrabe and Hrabetova, 2019), and suggest that the main mechanism for hypoxia speedup is also reduced *α*_ECS_.

We modeled SD in brain slices as a reaction-diffusion system in an unconnected neuronal population. Although we initiated SD by elevating extracellular K^+^ and tracked the position of K^+^ waves, the model itself was agnostic as to the agent of SD propagation. In our simulations of HSD, we saw that the initial elevation of extracellular K^+^ did not immediately initiate a spreading wave of K^+^, but led to delayed spread (Fig. 5*A*, Movie 2). In our simulations, as in the slice experiment, when K^+^ was applied to the tissue or hypoxia was induced, the elevation of K^+^ preceded the wavefront of SD-associated neuronal spiking. By contrast, when SD has been experimentally initiated in other ways, depolarization may precede, rather than follow, the K^+^ wave (Somjen, 2001). Incorporating synaptic connections into the model will provide a saltatory forward activation that will tend to speed up depolarization relative to the K^+^ wave; the balance of these factors will depend strongly on cell excitability, and the densities and lengths of connections.

### Model limitations

An irony of this type of study is that we are computer modeling an *in vitro* model of an *in vivo* animal model of human pathophysiology (model of a model of a model). There are necessarily distortions at each step. At the computer-modeling level, the major limitations of this study are the limitations that are inherent in all computer modeling—we necessarily made choices as to what to include and what to leave out. In the current set of simulations, we left out the following: (1) all neural connectivity; (2) types of neurons, including distinction between inhibitory and excitatory neurons; (3) dendritic morphologies plus additional membrane mechanisms; (4) glia, except as a generalized field; (5) volume-occupying structures (instead using fractional volumes); and (6) intracellular handling of ions and second messengers with effects on pumps and other mechanisms. These are largely correctable limitations that we will gradually begin to address in future versions of the model. Some, however, represent limitations in experimental knowledge that need to be addressed.

Additionally, we purposely designed the model to be generic rather than to reproduce the properties of any particular brain region and species. We feel this allowed us to generalize more readily (e.g., comparing SD in brainstem vs in cortex). Several interdependent tissue properties were treated independently with the benefit of allowing additional investigation by isolating parameters.

A major limitation of the model was the simplification in which Na^+^/K^+^ pumps consumed O_2_ to drive their conductances rather than ATP (Wei et al., 2014). This was partly justified by Na^+^/K^+^ pumps using 91% of available ATP in the brain under normal physiological conditions (Lennie, 2003); also, neurons synthesize ATP as needed (Davis, 2020). Because of ATP reserves in the tissue and the simplification using O_2_ as a proxy for ATP, the spread of hypoxia during SD in perfused slices may be slower than predicted by our simulations, but we still expect it to precede the SD wavefront *in vitro*.

We also note that extracellular Na^+^ concentration shifts following SD were smaller than observed experimentally, and intracellular concentration shifts were larger than expected (Somjen, 2001). This may reflect biophysical mechanisms absent from the model or a lack of appropriate volume changes in ECS and intraneuronal spaces. In particular, we did not model large, intracellular, negatively charged macromolecules that produce Gibbs–Donnan effects, which contribute to SD (Lemale et al., 2022).

### Experimentally testable predictions

Several of our predictions relate to the effects of manipulations on SD speed. These effects could be most easily assessed electrophysiologically by using a series of extracellular electrodes in a slice to note the time of population burst passage and subsequent time of silence (the depression phase).

#### Slower SD in brainstem slice compared with cortical slice

Compared with cortex, brainstem has lower cell density, higher *α*_ECS_, and low expression of ECS matrix molecules/perineural nets, implying low *λ*_ECS_ (Ogawa et al., 1985; Hobohm et al., 1998; Syková and Nicholson, 2008). All of these factors contribute to slower propagation speeds in our model (Figs. 5*C*, 6) Our analysis of principal cell morphologies from brainstem also suggested an S/V lower than those of neocortical principal cells, another factor contributing to slowing.

#### Increased SD speed with cytotoxic edema in penumbra after stroke or TBI

Cytotoxic edema will increase *β*_nrn_, producing speedup, which will be enhanced in the hypoxic condition (Fig. 6*B*). We note that some of the fastest SD speeds (∼9 mm/min) have been observed in patients after stroke (Woitzik et al., 2013).

#### Hypoxia will increase SD speed under multiple conditions

SD spread faster in hypoxic than in perfused slices. Experimentally, one would assess as follows: (1) remove O_2_ from bath; (2) monitor *α*_ECS_ to determine the onset of preanoxic depolarization phase (*α*_ECS_, ∼0.099–0.0179, depending on brain region; Pérez-Pinzón et al., 1995); and (3) add a K^+^ bolus. The slice should not be reperfused, unlike the procedure for HSD experiments (Pérez-Pinzón et al., 1995; Aitken et al., 1998).

#### Increasing *α*_ECS_ will attenuate SD propagation

This could be assessed using hypertonic saline to increase *α*_ECS_ (Kume-Kick et al., 2002). Hypertonic saline is sometimes used in reducing intracranial pressure after TBI (Oddo et al., 2009; Kamel et al., 2011; Mangat et al., 2020; Shi et al., 2020), and might therefore also reduce SD in these patients.

#### SD speed will be reduced by antiepileptic drugs

We showed here that dynamic changes in *α*_ECS_ due to SD itself speeds up the SD wave (Fig. 5*A*,*B*). Similar changes in *α*_ECS_ have been seen with ictal phenomena in the study by Colbourn et al. (2021), allowing us to hypothesize that this may be the linkage between SD and *α*_ECS_, presumably mediated through excessive release of neurotransmitters whether classical, peptidergic, or nitric oxide.

#### SD velocity will correlate with neural density, dendritic complexity, and total neuronal volume across regions

Measurements of SD in various brain regions and across species can be assessed. Density is determined with counts in Nissl stain. Dendritic complexity increases S/V and can be assessed on traced, biocytin-filled cells with measures such as we performed here. Total neuronal volume can be assessed by measuring ascorbic acid in tissue (Rice and Russo-Menna, 1997). These effects could also be evaluated in tissue culture or in organoids.

Increasing the diameter or concentration of the K^+^ bolus used to initiate SD beyond their thresholds will not change SD speed (Fig. 3).

#### Ease of SD initiation and SD propagation speed will increase with increasing slice thickness due to relatively hypoxic core

SD will be difficult or impossible to initiate in very thin slices (Fig. 7).

#### SD propagates through the core of the slice

Neurons near the surface of a perfused slice will be resistant to SD due to the high availability of O_2_ (Fig. 8). Extracellular recordings looking for bursting and subsequent depression at different slice depths could be performed to confirm this prediction. However, one may want to avoid measurement directly at the slice surface where neurons will have suffered damage due to slicing and therefore may exhibit additional pathology that could alter SD propagation.

### Future directions

Our model incorporated quantitative data and simpler models from numerous sources and at multiple spatial scales to constitute a unified framework for simulating SD in brain slices. We propose the use of this framework as a community tool for researchers in the field to test hypotheses; guide the design of new experiments; and incorporate new physiological, transcriptomic, proteomic, or anatomic data into the framework. The open-source, branchable, versioned GitHub repository can provide a dynamic database for SD simulations or modeling brain slices in general.

## Synthesis

Reviewing Editor: William Stacey, University of Michigan Decisions are customarily a result of the Reviewing Editor and the peer reviewers coming together and discussing their recommendations until a consensus is reached. When revisions are invited, a fact-based synthesis statement explaining their decision and outlining what is needed to prepare a revision will be listed below. The following reviewer(s) agreed to reveal their identity: Jens Dreier, Ghanim Ullah.

This paper uses a computational-only approach to predict several physiological (potentially testable in the future) variables that are involved in spreading depression. The model makes five main predictions: 1. SD can be inhibited by enlarging ECS volume; 2. SD velocity will be greater in areas with greater neuronal density, total neuronal volume, or larger/more dendrites; 3. SD is all-or-none: initiating K+ bolus properties have little impact on SD speed; 4. Slice thickness influences SD due to relative hypoxia in the slice core, exacerbated by SD in a pathological cycle; and 5. Higher neuronal spike rates and faster spread will be observed in the core than the periphery of perfused slice during SD.

While there was interest in this model, and the authors did acknowledge many of the limitations of the approach, what was most concerning was that many of the parameters chosen and conclusions seem to conflict with prior experimental evidence. The concern is that this work might cement beliefs that have evidence to the contrary, without reconciling these differences. Some of the examples below are from several years ago, and perhaps could be replicated. Would your results motivate a re-look at those older experiments? Were the conditions different? Or do those old results disprove your predictions, uncovering critical flaws in the simulation? That really is the main crux of the concerns here.

1. “We simulated SD in both perfused and hypoxic slices. Our model confirms that SD speed is augmented by propionate and hypoxia and suggests that changing the ECS is the principle mechanism through which they influence SD.” And “Hypoxia will increase SD speed under multiple conditions”.

This contrasts with the true findings in hypoxia. Aitken et al. 1998 wrote the following: “Both events (normoxic and hypoxic SD) arose focally, after which a clear advancing wave front of increased reflectance and DC shift spread along the CA1 stratum radiatum and s. oriens. The rate of spread was similar: conduction velocity of normoxic SD was 8.73 +/- 0.92 mm/min (mean +/- SE) measured electrically and 5.84 +/- 0.63 mm/min measured optically, whereas (hypoxic) HSD showed values of 7.22 +/- 1.60 mm/min (electrical) and 6.79 +/- 0.42 mm/min (optical). When initiated in CA1, normoxic SD consistently failed to enter the CA3 region (7/7 slices) and could not be initiated by direct KCl injection in the CA3 region (n = 3). Likewise, the hypoxic SD-like optical signal showed onset in the CA1 region and halted at the CA1/CA3 boundary (9/9 slices)...”

"Once the K+ wave arrived however, the cells dumped large quantities of K+ into the ECS due to the burst and subsequent prolonged depolarization (note rapid depletion of [K+]i - Fig. 4B).”

In other words, Aitken et al. 1998 found that the rate of spread was similar during normoxic and hypoxic SD. We agree that it appears highly plausible (without experiments) that SD speed could be higher during hypoxia-and may have believed this to be true as well. However, this was not the case in real measurements. Maybe, these measurements should be reproduced, but the Somjen group which did these measurements is trustworthy. Many of their findings could be reproduced by others.

In all this, it is important to realize that the extracellular space (ECS) shrinks by about 70% during SD. Normally, the ECS makes ∼20% of the total volume. During SD, the ECS makes ∼6% of the total volume. Even if not a single potassium ion left the intracellular space, the extracellular potassium concentration would increase from ∼3 to ∼10 mM. I.e., not many potassium ions have to leave the cells to increase the extracellular potassium concentration from ∼3 to ∼50 mM which is measured during SD. The truth is that only a small quantity of K+ is dumped into the ECS during SD in contrast to the large quantity of Na+ dumped into the intraneuronal space. Plausibility considerations based on volume ratios and volume changes suggest that more than 10 times as many sodium ions are taken up into the cells during SD than potassium ions leave the cells. Correspondingly, very large numbers of chloride ions are also taken up into the cell, and this is measured accordingly (Hansen and Zeuthen, 1981; Kraig and Nicholson, 1978; Windmuller et al., 2005). These relationships are important and should be better explained. The central ion to understand SD may not be potassium but sodium. At least the sodium shifts are much larger than the potassium shifts in absolute terms. Also, during recovery, while it is not wrong that the sodium pump also restores extracellular potassium to normal, it is primarily activated by intracellular sodium and must remove much more sodium from the intracellular space than potassium from the ECS. Sodium should not be forgotten in all this. The driving force leading to SD is related to the sodium gradient rather than the potassium gradient (Lemale et al., 2022).

2. “The Na+/K+ pump activity which contributed to K+ elimination from the ECS in the core of the slice created a high demand for O2, exceeding the rate at which it could diffuse in from the bath. This resulted in much of the tissue becoming hypoxic before the arrival of the K+ wave (compare rapid fall-off of O2 in Fig. 4E to much slower rise of extracellular K+ in Fig. 4F). The rapid spread of O2 deficit explains the total pump failure at intermediate locations in the slice.”

The authors seem to forget that the tissue has an ATP reserve. In vivo and also in vitro, complete ischemia/hypoxia usually requires a minute or even longer before SD occurs. Even severe hypoxia does not imply an immediate lack of ATP. Moreover, we are unaware of evidence that tissue oxygen falls before the SD front in normal tissue. It typically falls behind the wave front. For example, compare figure 2 in (Piilgaard and Lauritzen, 2009) or the figures in (Takano et al., 2007). The measured findings seem to directly contradict your findings of tissue hypoxia preceding the wave front. In addition, hyperbaric oxygenation does not seem to have profound effects on SD (e.g. figure 5 in (Lindauer et al., 2010)). In real measurements, tissue oxygen seems to drop behind the wave front, if it drops at all. Under certain conditions, tissue oxygen can also increase in response to SD (examples are given in figure 2 (Balanca et al., 2017) and figure 1 (Major et al., 2021).

3 “Our results support Grafstein’s hypothesis that SD is primarily a reaction-diffusion process mediated by a regenerative K+ wave [Grafstein, 1956].”

Somjen wrote in his review in 2001 with regard to a hypothesis based on a possible propagation mechanism based on potassium (Somjen, 2001): “Yet another problem is that, at a given point in the tissue, [K+]o does not start to increase ahead of the ΔVo, as it should, if K+ were the agent of the propagation of SD”. Thus, the original recordings contradict this hypothesis and they have been independently reproduced by several groups. The voltage shift is only preceded by an increase in [K+]o when K+ is either applied artificially or hypoxia is induced which activates ATP-sensitive and G protein-dependent Ca2+-sensitive K+ channels long before it induces SD (Erdemli et al., 1998; Muller and Somjen, 2000; Revah et al., 2016). There are good arguments that a reaction-diffusion process is the basis of SD, but there are also good arguments against K+ as the neuroactive factor responsible for SD propagation. The most important one is the one formulated by Somjen above. Original recordings should not be neglected. Maybe, this must formulated more cautiously. It is likely that the neuroactive factor responsible for the reaction diffusion process is of a different nature than potassium.

4. In Figure 4C, it appears too extreme that the intracellular sodium concentration can increase to values of up to 80 mM during SD. The intracellular space is four times larger than the ECS. Even if you remove astrocytes, it is impossible that intracellular sodium increases to 80 mM. Plausibility suggests that it increases to 36 mM (Lemale et al., 2022) and true measurements suggest increases from 10 to between 20 and 30 mM (Gerkau et al., 2017). So, this part of the figure is not convincing and suggests that you did not sufficiently consider the volume ratio and volume changes between intracellular space and ECS. In this respect, you may also have a look at Figure 4 in (Somjen, 2001).

Minor comments

The following two statements are synonyms: “SD can be inhibited by enlarging ECS volume” and “SD velocity will be greater in areas with greater neuronal density, total neuronal volume, or larger/more dendrites”. Greater neuronal density result in low ECS volume, which would increase the propagation of SD as indicated by the first statement.  

Figure 4 legend: Remove one “shows” in the text “Supporting Movie 4-1 shows shows extracellular ion”.

L 197: Should “S:V = 0.02 μm” be “S:V = 0.02/μm”?  

L 198: “K+ wave speed also increased with increased βnrn while keeping S:V and cell density constant (Fig. 6B).” It is not clear how can the cell density remain constant when neuronal volume relative to the tissue volume is changing? This comment also applies to the statements that follow: “Cell density effects were less marked, whether through keeping surface area and volume constant (Fig. 6C), or keeping βnrn and S:V constant (Fig. 6D).”

Legend of figure 4: “Supporting Movie 4-1 shows shows extracellular ion and O 2 concentrations...”

"SD is difficult to detect in humans noninvasively [Zandt et al., 2015], ...”

The original papers should also be cited here: (Drenckhahn et al., 2012; Hartings et al., 2014; Hofmeijer et al., 2018).

"SD has been studied in brain slices from a wide range of species and brain regions, including neocortex, hippocampus, brainstem, and retina... It can be triggered experimentally by various means, including electrical stimulation; mechanical insult; K+, glutamate, and ouabain application [Cozzolino et al., 2018].”

There is an important error in this sentence, which is a common misunderstanding and should be eradicated. Bath exposure to glutamate (up to 1 mM, which is pathophysiological) or glutamate receptor agonists induces depolarization and a negative direct current (DC) shift wherever one chooses to record in a brain slice. Imaging light transmission (LT) changes in an entire slice shows that this depolarization arises in the majority of imaged gray matter almost simultaneously, i.e. the depolarization does not spread throughout neocortical and hippocampal gray matter. A range of concentrations of bath-applied glutamate (0.01 to 1.0 mM), NMDA, or kainate fails to induce a “spreading” depolarization (Andrew et al., 1996; Andrew et al., 1999; Obeidat and Andrew, 1998). So, the elevated LT signal evoked by glutamate or by NMDA (denoting cell swelling and depolarization (Rungta et al., 2015)) develops uniformly in the slice and does not propagate. This contrasts with bath exposure to high K+, ouabain, hypoxia or oxygen-glucose deprivation which produce a true “spreading” depolarization (Aitken et al., 1998; Joshi and Andrew, 2001; Major et al., 2017; Petzold et al., 2005). Thus, glutamate should be removed from the list above.

"This mirrors findings in vivo where SD-associated metabolic demand for O2 can exceed supply, resulting in tissue hypoxia [Takano et al., 2007].”

The following references could be added for rats and humans (Piilgaard and Lauritzen, 2009; Winkler et al., 2017), but again your traces do not mirror the traces in real experiments where the drop in tissue oxygen follows the SD wave front.

The figure legends should have better descriptions of what is happening in the legends-they are too short. Non-modelling people should also be able to easily understand what is shown in the figures. Help the reader with brief summaries of what is shown. If more people understand what the authors want to explain in the figure, more people will cite this paper.

References cited above:

Aitken PG, Tombaugh GC, Turner DA, Somjen GG. Similar propagation of SD and hypoxic SD-like depolarization in rat hippocampus recorded optically and electrically. J Neurophysiol 1998; 80: 1514-21.

Akerman S, Holland PR, Goadsby PJ. Mechanically-induced cortical spreading depression associated regional cerebral blood flow changes are blocked by Na+ ion channel blockade. Brain Res 2008; 1229: 27-36.

Andrew RD, Adams JR, Polischuk TM. Imaging NMDA- and kainate-induced intrinsic optical signals from the hippocampal slice. J Neurophysiol 1996; 76: 2707-17.

Andrew RD, Jarvis CR, Obeidat AS. Potential sources of intrinsic optical signals imaged in live brain slices. Methods 1999; 18: 185-96, 79.

Balanca B, Meiller A, Bezin L, Dreier JP, Marinesco S, Lieutaud T. Altered hypermetabolic response to cortical spreading depolarizations after traumatic brain injury in rats. J Cereb Blood Flow Metab 2017; 37: 1670-86.

Drenckhahn C, Winkler MK, Major S, Scheel M, Kang EJ, Pinczolits A, et al. Correlates of spreading depolarization in human scalp electroencephalography. Brain 2012; 135: 853-68.

Erdemli G, Xu YZ, Krnjevic K. Potassium conductance causing hyperpolarization of CA1 hippocampal neurons during hypoxia. J Neurophysiol 1998; 80: 2378-90.

Gerkau NJ, Rakers C, Petzold GC, Rose CR. Differential effects of energy deprivation on intracellular sodium homeostasis in neurons and astrocytes. J Neurosci Res 2017; 95: 2275-85.

Hansen AJ, Zeuthen T. Extracellular ion concentrations during spreading depression and ischemia in the rat brain cortex. Acta Physiol Scand 1981; 113: 437-45.

Hartings JA, Wilson JA, Hinzman JM, Pollandt S, Dreier JP, DiNapoli V, et al. Spreading depression in continuous electroencephalography of brain trauma. Ann Neurol 2014; 76: 681-94.

Hofmeijer J, van Kaam CR, van de Werff B, Vermeer SE, Tjepkema-Cloostermans MC, van Putten M. Detecting Cortical Spreading Depolarization with Full Band Scalp Electroencephalography: An Illusion? Front Neurol 2018; 9: 17.

Joshi I, Andrew RD. Imaging anoxic depolarization during ischemia-like conditions in the mouse hemi-brain slice. J Neurophysiol 2001; 85: 414-24.

Kraig RP, Nicholson C. Extracellular ionic variations during spreading depression. Neuroscience 1978; 3: 1045-59.

Lemale CL, Luckl J, Horst V, Reiffurth C, Major S, Hecht N, et al. Migraine Aura, Transient Ischemic Attacks, Stroke, and Dying of the Brain Share the Same Key Pathophysiological Process in Neurons Driven by Gibbs-Donnan Forces, Namely Spreading Depolarization. Frontiers in cellular neuroscience 2022; 16: 837650.

Luckl J, Lemale CL, Kola V, Horst V, Khojasteh U, Oliveira-Ferreira AI, et al. The negative ultraslow potential, electrophysiological correlate of infarction in the human cortex. Brain 2018; 141: 1734-52.

Major S, Petzold GC, Reiffurth C, Windmuller O, Foddis M, Lindauer U, et al. A role of the sodium pump in spreading ischemia in rats. J Cereb Blood Flow Metab 2017; 37: 1687-705.

Muller M, Somjen GG. Na(+) and K(+) concentrations, extra- and intracellular voltages, and the effect of TTX in hypoxic rat hippocampal slices. J Neurophysiol 2000; 83: 735-45.

Obeidat AS, Andrew RD. Spreading depression determines acute cellular damage in the hippocampal slice during oxygen/glucose deprivation. Eur J Neurosci 1998; 10: 3451-61.

Petzold GC, Windmuller O, Haack S, Major S, Buchheim K, Megow D, et al. Increased extracellular K+ concentration reduces the efficacy of N-methyl-D-aspartate receptor antagonists to block spreading depression-like depolarizations and spreading ischemia. Stroke 2005; 36: 1270-7.

Piilgaard H, Lauritzen M. Persistent increase in oxygen consumption and impaired neurovascular coupling after spreading depression in rat neocortex. J Cereb Blood Flow Metab 2009; 29: 1517-27.

Revah O, Lasser-Katz E, Fleidervish IA, Gutnick MJ. The earliest neuronal responses to hypoxia in the neocortical circuit are glutamate-dependent. Neurobiol Dis 2016; 95: 158-67.
